# In ovo feeding of α-ketoglutaric acid improves hepatic antioxidant-gene expression, plasma antioxidant activities and decreases body temperature without affecting broiler body weight under cyclic heat stress

**DOI:** 10.1016/j.psj.2024.103749

**Published:** 2024-04-09

**Authors:** Vaishali Gupta, Chris Major Ncho, Akshat Goel, Chae-Mi Jeong, Yang-Ho Choi

**Affiliations:** ⁎ Department of Animal Science, Gyeongsang National University, Jinju 52828, Republic of Korea; † Division of Applied Life Sciences (BK21 Four Program), Gyeongsang National University, Jinju 52828, Republic of Korea; ‡ Institute of Agriculture and Life Sciences, Gyeongsang National University, Jinju 52828, Republic of Korea

**Keywords:** broiler, in ovo feeding, AKG, gene expression, heat stress

## Abstract

The broiler industry is adversely affected by the rise in global temperature. This study investigated the effects of in ovo feeding of α-ketoglutaric acid (**AKG**) on growth performance, organ weight, plasma metabolite, plasma oxidative stress, rectal temperature (**RT**), and hepatic mRNA expression of antioxidant-related genes in Arbor Acres broilers subjected to cyclic heat stress (**HS**). Three hundred fifty fertile eggs during incubation were divided into 5 groups according to AKG concentrations and temperature conditions. After dissolving AKG in distilled water at 0, 0.5, 1.0, and 1.5, 0% AKG was in ovo administered to 2 of the 5 groups whereas the remaining 3 groups received 0.5, 1.0, and 1.5%, respectively. From d 29 to 34 of age, 4 groups of birds received heat stress (**HS**) at 31°C ± 1°C for 6 h per day while the other group was kept at room temperature (21°C ± 1°C; NT). So, the 5 treatment groups were: 1) 0AKG-NT, where chicks hatched from eggs receiving 0% AKG were reared under thermoneutral conditions. 2) 0AKG-HS, where chicks hatched from eggs receiving 0% AKG were reared under cyclic HS conditions. 3) 0.5AKG-HS, where chicks hatched from eggs receiving 0.5% AKG were reared under cyclic HS conditions. 4) 1.0AKG-HS, where chicks hatched from eggs receiving 1.0% AKG were reared under cyclic HS conditions. 5) 1.5AKG-HS, where chicks hatched from eggs receiving 1.5% AKG were reared under cyclic HS conditions. HS significantly reduced body weight change (ΔBW %) and average daily gain (**ADG**) without affecting average daily feed intake (**ADFI**). Feed conversion ratio (**FCR**) was significantly increased (*P* = 0.003) in all HS-treated groups. A significant linear decrease in the final RT (*P* = 0.005) and a change in RT (*P* = 0.003) were detected with increasing AKG concentration. Total antioxidant capacity (*P* = 0.029) and antioxidant balance (*P* = 0.001) in plasma increased linearly with increasing AKG concentration whereas malondialdehyde concentrations were linearly decreased (*P* = 0.001). Hepatic gene expression of CAT (*P* = 0.026) and GPX1 (*P* = 0.001) were dose-dependently upregulated while nicotinamide adenine dinucleotide phosphate oxidase (**NOX**)1, NOX4, and heat shock protein (HSP)70 were linearly downregulated (*P* < 0.05). Hence, in ovo injection of AKG was effective in mitigating HS-induced oxidative stress without attenuating the adverse effects on broiler growth.

## INTRODUCTION

Escalating global temperature makes heat stress (**HS**) one of the major menaces of the livestock industry (He et al., 2018). When there is an imbalance in the amount of heat generated and heat dissipated from the body, the organism may be under HS (Nawab et al., 2018). Numerous external factors influence the extent of HS, including global temperature, geographical location, weather conditions, and the time of the day. Intrinsic factors such as genetic predisposition, sex of the animal, rate of metabolism, and the presence or absence of thermoregulatory mechanisms are also associated with HS (Lara and Rostagno, 2013). The present-day broiler strains are a result of an extensive genetic selection achieved through years for higher yield and productivity (Hunton, 2006). In fact, a 400% increase in growth rates in less than 5 decades (1957–2005) has been witnessed (Zuidhof et al., 2014). Genetic selection has been a major player contributing over 85 to 90% and the remainder has been attributed to diet (Havenstein et al., 2003). Although extensive selection increased body weight yields and improved FCR, it also significantly curtailed the immune response of broilers over time (Cheema et al., 2003). Genetic improvements in growth have been associated with health-related problems in modern broilers. Immune function is negatively associated with growth and carcass characteristics. Selection for improved growth and carcass traits resulted in broilers that are refractory to immunological stress and are susceptible to infections. It is noteworthy to mention here that it is inequitable to expect broilers to be bred solely for higher immunocompetence at the expense of growth and production indices. Further, the lack of sweat glands makes poultry birds more susceptible to heat stress (Ncho et al., 2021a).

In birds, HS adversely affects their behavior (Mack et al., 2013; Li et al., 2015; Rath et al., 2015), physiology (Awad et al., 2020), plasma biochemistry (Luo et al., 2018; Hosseini-Vashan et al., 2020), immunity (Niu et al., 2009; Hosseini-Vashan et al., 2020), oxidative balance (Ncho et al., 2021a), production parameters (Imik et al., 2012), and meat quality (Smith, 1993; Lu et al., 2007). Hence, multidimensional effects have been made over the years to mitigate the inimical effects of HS on broilers (Mahmoud and Edens, 2003; Sohail et al., 2012; Hu et al., 2020a; Ncho et al., 2021a; Goel et al., 2022; Goel et al., 2023). In ovo delivery system of substances could be one such method that can be explored to develop thermotolerance in broilers of marketable age. In ovo feeding of galacto-oligosaccharides, for example, not only mitigated the deleterious effects of HS but also helped improve feed efficiency during the finishing stage of broilers (Slawinska et al., 2020). Improved activity of antioxidant enzymes was reported by an in ovo supplementation of vitamin E and nanocurcumin (Heidary et al., 2020). Our previous studies have also shown that feeding ᵞ-aminobutyric acid (GABA) in ovo can improve thermotolerance in broilers (Ncho et al., 2021a; Ncho et al., 2021b; Ncho et al., 2022a).

It is well established that α-ketoglutaric acid (**AKG**) is a keto-acid that is biosynthesized when oxaloacetate or glucose reacts with pyruvate (Harrison and Pierzynowski, 2008), acts as the speed-determinative factor in the citric acid cycle (Wu et al., 2016), and combines with hydrogen peroxide (**H_2_O_2_**) to form succinate, carbon dioxide, and water (Long and Halliwell, 2011). Hence, it not only acts as an energy producing compound but is also a potent antioxidant. In the body, hepatocytes and enterocytes convert AKG to glutamine, which is further used for ATP production (Pierzynowski and Sjodin, 1998). Dietary AKG resulted in reduced malondialdehyde (**MDA**) and H_2_O_2_ concentrations in the liver and increased jejunal glutathione peroxidase (**GPX1**) and ileal catalase (**CAT**) activities in Cherry Valley ducks (Guo et al., 2017). Dietary supplementation of AKG improved intestinal energy levels in ducks (Guo et al., 2017) and meat quality in layers (Tomaszewska et al., 2020).

Hypothesizing that in ovo supplementation of AKG can effectively mitigate the adverse effects caused due to HS in broilers, this study was designed to determine the effects of in ovo feeding of AKG on broilers of marketable age exposed to cyclic HS for 6 d.

## MATERIALS AND METHODS

All the relevant procedures were approved by the Animal Care and Use Committee of Gyeongsang National University (GNU-200916-C0058).

### Egg Incubation and In Ovo Protocol

Eggs laid by 40-wk-old Arbor Acres hens were procured from a commercial local farm (Harim hatchery, Iksan, South Korea). After weighing and labeling the eggs individually, they were transferred to the egg incubator (Rcom Co., Ltd., Kimhae, Korea). Embryonic development in the eggs was tested at embryonic d 10 (ED10) by candling to remove infertile eggs. At ED17, a second candling was performed to remove any other remaining infertile eggs. Finally, 350 eggs were selected and distributed in 4 groups.

At ED17.5, these groups received an in ovo injection of AKG dissolved in distilled water (DDW) at 0% (n = 140 eggs), 0.5% (n = 70 eggs), 1.0% (n = 70 eggs), and 1.5% (n = 70 eggs). A 0.6 mL solution (w/v) of AKG (#75890, Sigma-Aldrich, St. Louis, MO) was prepared and injected into the eggs according to their treatment groups as described in our previous study (Ncho et al., 2021b). Briefly, a small area towards the broad end of the eggs was disinfected with 70% ethyl alcohol, and later, a tiny hole was drilled with the help of a dental drill (Saeshin, Daegu, Korea). A syringe 23-G needle 1-inch in length was inserted in the egg and 0.6 mL solution was injected into the amnion. Finally, a surgical tape (3M Micropore, Saint Paul, MO) was used to seal the hole.

### Rearing and Management of the Birds

The birds hatched were raised in battery cages with a brooding temperature of 36°C ± 1°C at 50 ± 5% relative humidity (**RH**). Once every other day, the temperature was reduced by 1°C to reach 21°C ± 1°C, 50 ± 5% (RH) at d 28 of rearing. A total of 6 cages (6 birds per cage) per treatment group were employed for the trial and each cage was considered as 1 replicate. Each cage measured 90 cm × 70 cm × 45 cm (length × width × height). Thus, the stocking density being 1,050 cm^2^ per bird at the day of hatch. During the first week of age, they were fed with commercial starter ration followed by commercial grower ration for the next 2 wk, and finally with commercial finisher ration from d 22 of age. The birds received water and feed (Nonghyup Feed, Gyeongju, Korea, supplementary file1) ad libitum.

Starting from d 29, 4 groups of birds were subjected to cyclic HS at 31°C ± 1°C for 6 h/d (HS) for 6 consecutive days while the other group was kept at standard rearing temperature (21°C ± 1°C; NT). Each day, the cyclic HS was scheduled from 9 am to 3 pm. During this phase, the broilers were reared under a lighting regime of 23 h light + 1 h dark (Chen et al., 2013). The lights were scheduled to switch off at 11 pm until midnight. A total of 180 birds were distributed into 1 of 5 treatment groups based on AKG dose and rearing temperature. 1) 0AKG-NT, where chicks hatched from eggs receiving 0% AKG were reared under thermoneutral conditions (negative control). 2) 0AKG-HS, where chicks hatched from eggs receiving 0% AKG were reared under cyclic HS conditions (positive control). 3) 0.5AKG-HS, where chicks hatched from eggs receiving 0.5% AKG were reared under cyclic HS conditions. 4) 1.0AKG-HS, where chicks hatched from eggs receiving 1.0% AKG were reared under cyclic HS conditions. 5) 1.5AKG-HS, where chicks hatched from eggs receiving 1.5% AKG were reared under cyclic HS conditions.

### Recording Growth Performances

Body weight (**BW**) and feed intake (**FI**) were recorded for the HS period. The BW initial was recorded before starting the HS experiment on d 29, whereas the BW final was recorded on d 34 after completion of the HS treatment. The difference/change in BW (ΔBW) was calculated as:

ΔBW(%)=((BWfinal−BWinitial)/BWinitial)×100

Average daily gain (ADG) gram/bird was calculated as:

ADG(g/bird)=(BWfinal−BWinitial)/totalnumberofHSdays

Average daily feed intake (ADFI)/bird was calculated as:

ADFI=(TotalfeedofferedduringtheHSperiod−feedremaininginthefeedersaftercompletionofHSperiod)/totalnumberofHSdays

Feed conversion ratio (FCR) was calculated as:

FCR=(feedconsumed/ΔBW)

### Collection of Samples

After the completion of cyclic HS, 6 birds were selected randomly from each treatment for blood and tissue collection. After the birds were euthanized using a carbon dioxide chamber, blood was collected directly into heparinized vacuum containers (#367874, BD Co., Ltd., Franklin Lakes, NJ) via heart puncture, and then centrifuged at 2,000 × *g* for 10 min at 4°C to separate and collect plasma which was stored at −20°C. Organs were excised free from the body and weighed for the liver, spleen, and bursa of Fabricius. Immediately after collection, liver samples were snap-frozen in liquid nitrogen, and stored at −80°C for later use. Relative organ weight (%) was calculated as:

Relativeorganweight(%)=(absoluteorganweight/bodyweightofthebird)×100

### Plasma Metabolite Concentration and Antioxidative Capacity

A VetTest Chemistry Analyzer (IDEXX Co., Ltd., Westbrook, ME) was used to determine concentrations of glucose, total protein, triglycerides, cholesterol, alanine transaminase (**ALT**), aspartate aminotransferase (**AST**), calcium (**Ca**), albumin, and globulin in plasma. Plasma total antioxidative capacity (**TAC**) was measured via 2,2-diphenyl-1-picrylhydrazyl–radical scavenging activity assay (**DPPH-RSA**) as previously described (Gerasopoulos et al., 2015). Briefly, 20 µL of plasma was added to 480 µL of 10 mmol/L sodium-potassium phosphate solution (pH 7.4). Next, an equal volume of 0.1 mmol/L of DPPH reagent (# 1898-66-4, Thermo Fisher Scientific, MA) was mixed into the diluted plasma sample and incubated for 30 min in the dark. Finally, the solution was centrifuged at 10,000 × *g* for 6 min and the absorbance was read at 517 nm. A solution was prepared using 500 µL of a 0.1 mmol/L DPPH reagent and 480 µL of a 10 mmol/L sodium-potassium phosphate solution and used as a negative control. Inhibitory activity was expressed in percentage using the formula:

DPPH(%inhibition)=[1−(A1/A0)]×100,

where A0 is the absorbance of the control and A1 is the absorbance of the test sample.

MDA concentration was measured as per a previously described protocol (Jyothi et al., 2008). Briefly, 400 µL of 40% trichloroacetic acid (TCA; # 76-03-9, Merck, Sigma-Aldrich, St. Louis, MO) was taken in a tube and an equal volume of plasma was added to it. Further, 800 µL of 0.67% thiobarbituric acid (TBA; # 504-17-6, Merck, Sigma-Aldrich, St. Louis, MO) was added to the tube and thoroughly vortexed. The tubes were then placed in a water bath at 95°C for 45 min followed by cooling on ice for 5 min. The samples were then centrifuged at 1,000 × g for 6 min and lastly, absorbance was read at 520 nm. The final concentration of MDA was estimated by the formula:

$$MDA\;concentration\left(molL^{-1}\right)=A/\left(Kxh\right),$$

where A is the absorbance of the sample; K is the molar extinction coefficient (1.5 × 10^5^ liter mol^−1^ cm^−1^); and h is the length of the cuvette used (1 cm).

Antioxidant balance was calculated as the ratio of DPPH-RSA and MDA values as previously described (Ncho et al., 2021a).

### Real-Time Polymerase Chain Reaction for mRNA Quantification

As described in our previous studies (Gupta et al., 2022; Ncho et al., 2023a), total RNA was extracted from 50 mg liver tissue using TRIzol(TM) reagent (# 15596018, Thermo Fisher Scientific, Waltham, MA) following the manufacturer's guide. Concentrations and purity of RNA samples were measured as the ratio of optical densities at 260 and 280 nm using a NanoDrop 2000 Spectrophotometer (pedestal mode, Thermo Scientific). Further, cDNA was synthesized from the RNA samples using Verso cDNA Synthesis Kit (# AB1453A, Thermo Fisher) following the manufacturer's guide and was stored at −20°C. Different genes were amplified using a StepOnePlus real-time PCR system (Life Technologies, Carlsbad, CA). Forward and reverse primers (10 pmol) of specific genes and 10 µL Power SYBR green PCR master mix (Life Technologies) were included in each reaction along with the cDNA to make a total volume of 20 µL. The primer sequences of the genes used in the present study are presented in Table 1. Two housekeeping genes employed were GAPDH and β-ACTIN, and their Ct values were used to normalize the target genes' quantification. As previously described, fold change (FC) was determined using the 2^–ΔΔCt^ algorithm (Livak and Schmittgen, 2001), and gene expression was evaluated as log_2_ FC (Pietrzak et al., 2020).

**Table 1:** Oligonucleotide primer sequence for RT-qPCR.

No.	Gene	Sequence	Accession number	Reference
1.	GAPDH	F: TTGGCATTGTGGAGGGTCTTA R: GTGGACGCTGGGATGATGTT	NM_204305.1	(Goel et al., 2022)
2.	β-actin	F: ACCGGACTGTTACCAACA R: GACTGCTGCTGACACCTT	NM_205518.1	(Zhang et al., 2018)
3.	NRF2	F: CAGAAGCTTTCCCGTTCATAGA R: GACATTGGAGGGATGGCTTAT	NM_205117	(Zhang et al., 2018)
4.	CAT	F: ACCAAGTACTGCAAGGCGAA R: TGAGGGTTCCTCTTCTGGCT	NM_001031215.1	(Goel et al., 2022)
5.	SOD	F: AGGGGGTCATCCACTTCC R: CCCATTTGTGTTGTCTCCAA	NM_205064.1	(Goel et al., 2022)
6.	GPX1	F: AACCAATTCGGGCACCAG R: CCGTTCACCTCGCACTTCTC	NM_001277853.2	(Ncho et al., 2021b)
7.	HSP70	F: GCTGAACAAGAGCATCAATCCA R: CAGGAGCAGATCTTGCACATTT	AY143693.1	(Goel et al., 2022)
8.	HSP90	F: CCCGAGCAAGCTGGATTCT R: GGTCATCCCTATGCCGGTATC	NM_001109785	(Goel et al., 2022)
9.	NOX1	F: GCGAAGACGTGTTCCTGTAT R: GAACCTGTACCAGATGGACTTC	NM_001101830.1	(Ncho et al., 2023a)
10.	NOX4	F: CCTCTGTGCTTGTACTGTGTAG R: GACATTGGAGGGATGGCTTAT	NM_001101829.1	(Ncho et al., 2023a)

Abbreviations: GAPDH, Glyceraldehyde-3-Phosphate Dehydrogenase; β-actin, Beta-actin; NRF2, Nuclear factor erythroid 2-related factor; CAT, Catalase; SOD, Superoxide Dismutase; GPX1, Glutathione Peroxidase 1; HSP70, Heat Shock Protein 70; HSP90, Heat Shock Protein 90; NOX1, Nicotinamide adenine dinucleotide phosphate oxidase 1; NOX4, Nicotinamide adenine dinucleotide phosphate oxidase 4.

### Statistical Analysis

Growth performance, rectal temperature (**RT**), organ weights, plasma metabolites, plasma antioxidative capacity, and hepatic mRNA expression were analyzed using ANOVA, followed by Tukey's post-hoc test to determine differences between means (*P* < 0.05). To estimate dose-related effects, polynomial regression analysis was performed in the absence of the 0AKG-NT group. Results are presented as mean ± SEM.

A hierarchically clustered heatmap was created for the relative gene expression data to detect possible patterns. Multivariate analysis for hepatic mRNA expression of various HS-related genes was also performed via principle component analysis (**PCA**). To have a better contrast on the graphs, treatments (0 AKG-NT, 0AKG-HS, 0.5 AKG-HS, 1.0 AKG-HS, and 1.5 AKG-HS), challenges (NT vs. HT), and solutions (DDW vs. AKG) were used as supplementary variables.

Polynomial regression analysis, Pearson's correlation analysis, and one-way ANOVA were conducted using IBM SPSS Statistics for Windows software (IBM SPSS 27; IBM Corp., Armonk, NY). The “ComplexHeatmap” and “FactoMineR” packages of the R software version 4.0.3 (R Core Team 2020, R Foundation for Statistical Computing, Vienna, Austria) were used to execute the hierarchical clustering and PCA, respectively. Graphs were constructed using Graph Pad Prism 8 (GraphPad, La Jolla, CA).

## RESULTS

### Growth Performance and Organ Weights

Table 2 represents the growth parameters recorded during the cyclic HS period. The initial BW of the birds showed a quadratic (*P* = 0.024) increase in the BW of the birds as the dose of in ovo AKG was increased. Further, all the groups subjected to cyclic HS had a significantly lower BW (*P* = 0.004) as compared to 0AKG-NT, except for 1.5AKG-HS which was significantly indifferent. The percentage change in BW (ΔBW %) was significantly lower (*P* = 0.001) in the groups subjected to HS as compared to 0AKG-NT. Although the average daily feed intake (**ADFI**) was significantly indifferent among different treatment groups, the average daily gain (ADG, *P* = 0.001) and feed conversion ratio (FCR, *P* = 0.003) were also seen significantly lower in the groups subjected to HS compared to 0AKG-NT group. Mortality across all the groups was too low to deduce a statistical conclusion and hence was expressed in percentage (%) and numerically added in Table 2.

**Table 2:** Effect of in ovo administration of a graded dosage of AKG on the growth and mortality of broilers subjected to cyclic heat stress for 6 days from days 29 to 34.

Parameters	Treatments					*P*-value		
	0AKG-NT	0AKG-HS	0.5AKG-HS	1.0AKG-HS	1.5AKG-HS	ANOVA1	Lin2	Quad2
BW initial	1500.1 ± 32.8	1567.3 ± 29.5	1489.6 ± 38.2	1523.9 ± 36.1	1593.0 ± 21.2	0.123	0.475	0.024
BW final	1981.1 ± 34.9a	1853.5 ± 31.4b	1831.0 ± 30.0b	1836.7 ± 34.0b	1882.4 ± 20.7ab	0.004	0.500	0.250
ΔBW (%)	32.5 ± 1.3a	18.5 ± 1.1b	23.8 ± 2.1b	21.0 ± 1.5b	18.4 ± 1.5b	0.001	0.728	0.015
ADG (g/bird)	80.2 ± 2.7a	47.7 ± 2.7b	43.2 ± 5.4b	40.0 ± 4.6b	35.4 ± 4.7b	0.001	0.863	0.049
ADFI (g/bird)	119.3.0 ± 3.2	112.8 ± 1.7	114.0 ± 3.2	115.1 ± 3.1	111.1 ± 2.6	0.330	0.726	0.339
FCR	1.9 ± 0.1a	3.2 ± 0.2b	2.8 ± 0.1ab	3.0 ± 0.2b	3.3 ± 0.4b	0.003	0.603	0.142
Mortality (%)	3.33	3.33	6.67	3.33	6.67	NA	NA	NA

At 17.5 days of incubation, eggs were injected with 0.6mL of 0% (0AKG), 0.5% (0.5AKG), 1.0% (1.0AKG), and 1.5% (1.5AKG). At the 29th day of age, birds were subjected to heat stress at 31°C ± 1°C (HS) or not (NT) and were divided into 5 groups: 0AKG-NT, 0AKG-HS, 0.5AKG-HS, 1.0AKG-HS, and 1.5AKG-HS. BW initial was recorded before starting the heat stress experiment on Day 29. BW final was recorded after finishing the 6 days of heat stress on Day 34 Data are presented as mean ± SEM (n = 6).

Means bearing different superscripts differ significantly in a row (*P* < 0.05).

Abbreviations: BW, body weight; ΔBW, change in BW; ADG, average daily gain; ADFI, average daily feed intake; FCR, feed conversion ratio; Lin, linear effect; Quad, quadratic effect. 1p-value of all the treatment groups. 2p-value of all the treatment groups except the 0-AKG-NT.

The absolute and relative organ weights of the liver, spleen, and bursa of Fabricius did not differ significantly among different treatment groups (Table 3).

**Table 3:** Effect of in ovo administration of AKG on the absolute and relative organ weights of the liver, spleen, and bursa of Fabricius in broilers subjected to cyclic heat stress for 6 days from days 29 to 34.

Parameters	Treatments					*P*-value		
	0AKG-NT	0AKG-HS	0.5AKG-HS	1.0AKG-HS	1.5AKG-HS	ANOVA1	Lin2	Quad2
Absolute organ weight (g)								
Liver	47.9 ± 3.1	55.6 ± 2.7	50.1 ± 1.7	51.5 ± 2.9	49.3 ± 2.3	0.296	0.118	0.510
Spleen	2.3 ± 0.1	2.7 ± 0.1	2.7 ± 0.3	2.4 ± 0.2	2.3 ± 0.3	0.680	0.260	0.786
Bursa of Fabricius	2.6 ± 0.2	2.9 ± 0.2	3.1 ± 0.2	2.5 ± 0.2	2.9 ± 0.3	0.373	0.684	0.635
Relative organ weight (%)								
Liver	2.5 ± 0.1	2.6 ± 0.1	2.6 ± 0.1	2.5 ± 0.1	2.5 ± 0.1	0.763	0.245	0.816
Spleen	0.1 ± 0.0	0.1 ± 0.0	0.1 ± 0.0	0.1 ± 0.0	0.1 ± 0.0	0.548	0.306	0.443
Bursa of Fabricius	0.1 ± 0.0	0.1 ± 0.0	0.2 ± 0.0	0.1 ± 0.0	0.1 ± 0.0	0.179	0.931	0.903

At 17.5 days of incubation, eggs were injected with 0.6mL of 0% (0AKG), 0.5% (0.5AKG), 1.0% (1.0AKG), and 1.5% (1.5AKG). At the 29th day of age, birds were subjected to heat stress at 31°C ± 1°C (HS) or not (NT) and were divided into 5 groups: 0AKG-NT, 0AKG-HS, 0.5AKG-HS, 1.0AKG-HS, and 1.5AKG-HS.

Data are presented as Mean ± SEM (n = 6).

Means bearing different superscripts differ significantly in a row (*P* < 0.05).

Abbreviations: Lin, linear effect; Quad, quadratic effect. 1p-value of all the treatment groups. 2p-value of all the treatment groups except the 0AKG-NT.

### Rectal Temperature

There were no significant changes in the initial RT among treatment groups (Table 4). Regardless of AKG dosage, however, the final RT was significantly increased under HS while remaining constant in 0AKG-NT (*P* = 0.001). The change in RT (ΔRT) was significantly higher in all the treatment groups subjected to HS compared to 0AKG-NT. However, a linear decrease in the final RT (*P* = 0.005) and ΔRT (*P* = 0.003) was detected with increasing AKG dose under HS.

**Table 4:** Effect of in ovo administration of a graded dosage of AKG on the rectal temperature of broilers subjected to cyclic heat stress for 6 days from days 29 to 34.

Parameters	Treatments					*P*-value		
	0AKG-NT	0AKG-HS	0.5AKG-HS	1.0AKG-HS	1.5AKG-HS	ANOVA1	Lin2	Quad2
RT initial	41.5 ± 0.1	41.2 ± 0.0	41.3 ± 0.0	41.3 ± 0.0	41.4 ± 0.1	0.069	0.068	0.722
RT final	41.4 ± 0.0^a^	43.0 ± 0.1^d^	42.6 ± 0.1^bc^	42.8 ± 0.1^cd^	42.4 ± 0.1^b^	0.001	0.005	0.021
ΔRT	0.0 ± 0.1^a^	1.8 ± 0.1^c^	1.4 ± 0.1^bc^	1.6 ± 0.1^c^	1.1 ± 0.2^b^	0.001	0.003	0.753

At 17.5 days of incubation, eggs were injected with 0.6mL of 0% (0AKG), 0.5% (0.5AKG), 1.0% (1.0AKG), and 1.5% (1.5AKG). At the 29th day of age, birds were subjected to heat stress at 31°C ± 1°C (HS) or not (NT) and were divided into 5 groups: 0AKG-NT, 0AKG-HS, 0.5AKG-HS, 1.0AKG-HS, and 1.5AKG-HS. RT initial was recorded before starting the heat stress experiment on Day 29. RT final was recorded after finishing the 6 days of heat stress on Day 34 Data are presented as Mean ± SEM (n = 6). Means bearing different superscripts differ significantly in a row (*P* < 0.05).

Abbreviations: RT, rectal temperature; ΔRT, change in RT; Lin, linear effect; Quad, quadratic effect. 1p-value of all the treatment groups. 2p-value of all the treatment groups except the 0-AKG-NT.

### Plasma Metabolites Estimation

Table 5 shows plasma concentrations of glucose, total protein, triglycerides, cholesterol, ALT, AST, Ca, albumin, and globulin. Ca concentration was significantly higher in 1.0AKG-HS compared to 0AKG-HS (*P* = 0.009) and also changed quadratically under HS as the amount of in ovo AKG increased (*P* = 0.005).

**Table 5:** Effect of in ovo administration of a graded dosage of AKG on the plasma concentration of glucose, total protein, triglycerides, cholesterol, ALT, AST, calcium, albumin, and globulin of broilers subjected to cyclic heat stress for 6 days from days 29 to 34.

Parameters	Treatments					*P*-value		
	0AKG-NT	0AKG-HS	0.5AKG-HS	1.0AKG-HS	1.5AKG-HS	ANOVA1	Lin2	Quad2
Glucose (mg/dL)	260.8 ± 8.1	281.3 ± 6.7	273.5 ± 5.2	275.0 ± 7.7	268.3 ± 8.6	0.380	0.235	0.935
Total protein (g/dL)	3.0 ± 0.1	3.1 ± 0.1	3.1 ± 0.2	3.2 ± 0.2	3.1 ± 0.2	0.888	0.751	0.843
Triglycerides (mg/dL)	39.5 ± 10.2	25.3 ± 2.8	21.3 ± 1.7	31.2 ± 2.4	24.0 ± 3.9	0.137	0.675	0.617
Cholesterol (mg/dL)	120.0 ± 5.1	127.2 ± 5.6	121.2 ± 5.7	130.7 ± 7.3	132.2 ± 8.4	0.595	0.420	0.585
ALT (U/L)	22.0 ± 3.7	28.0 ± 3.8	21.5 ± 2.9	19.5 ± 3.3	19.8 ± 2.1	0.358	0.062	0.266
AST (U/L)	334.5 ± 40.6	196.5 ± 4.8	229.5 ± 23.7	280.8 ± 45.9	270.7 ± 45.2	0.103	0.080	0.530
Ca (mg/dL)	10.4 ± 0.1^ab^	9.8 ± 0.2^a^	10.3 ± 0.1^ab^	11.0 ± 0.4^b^	9.8 ± 0.2^a^	0.009	0.620	0.005
Albumin (g/dL)	1.4 ± 0.1	1.4 ± 0.0	1.4 ± 0.1	1.5 ± 0.1	1.4 ± 0.1	0.755	0.502	0.501
Globulin (g/dL)	1.6 ± 0.1	1.7 ± 0.1	1.7 ± 0.1	1.8 ± 0.1	1.7 ± 0.1	0.797	0.972	0.939

At 17.5 days of incubation, eggs were injected with 0.6mL of 0% (0AKG), 0.5% (0.5AKG), 1.0% (1.0AKG), and 1.5% (1.5AKG). On the 29th day of age, birds were subjected to heat stress at 31°C ± 1°C (HS) or not (NT) and were divided into 5 groups: 0AKG-NT, 0AKG-HS, 0.5AKG-HS, 1.0AKG-HS, and 1.5AKG-HS. Data are presented as Mean ± SEM (n = 6). Means bearing different superscripts differ significantly in a row (*P* < 0.05).

Abbreviations: ALT, alanine transaminase; AST, aspartate aminotransferase; Ca, calcium; Lin, linear effect; quad, quadratic effect. 1p-value of all the treatment groups. 2p-value of all the treatment groups except the 0-AKG-NT

### Plasma Antioxidative Capacity

**Figure 1:**
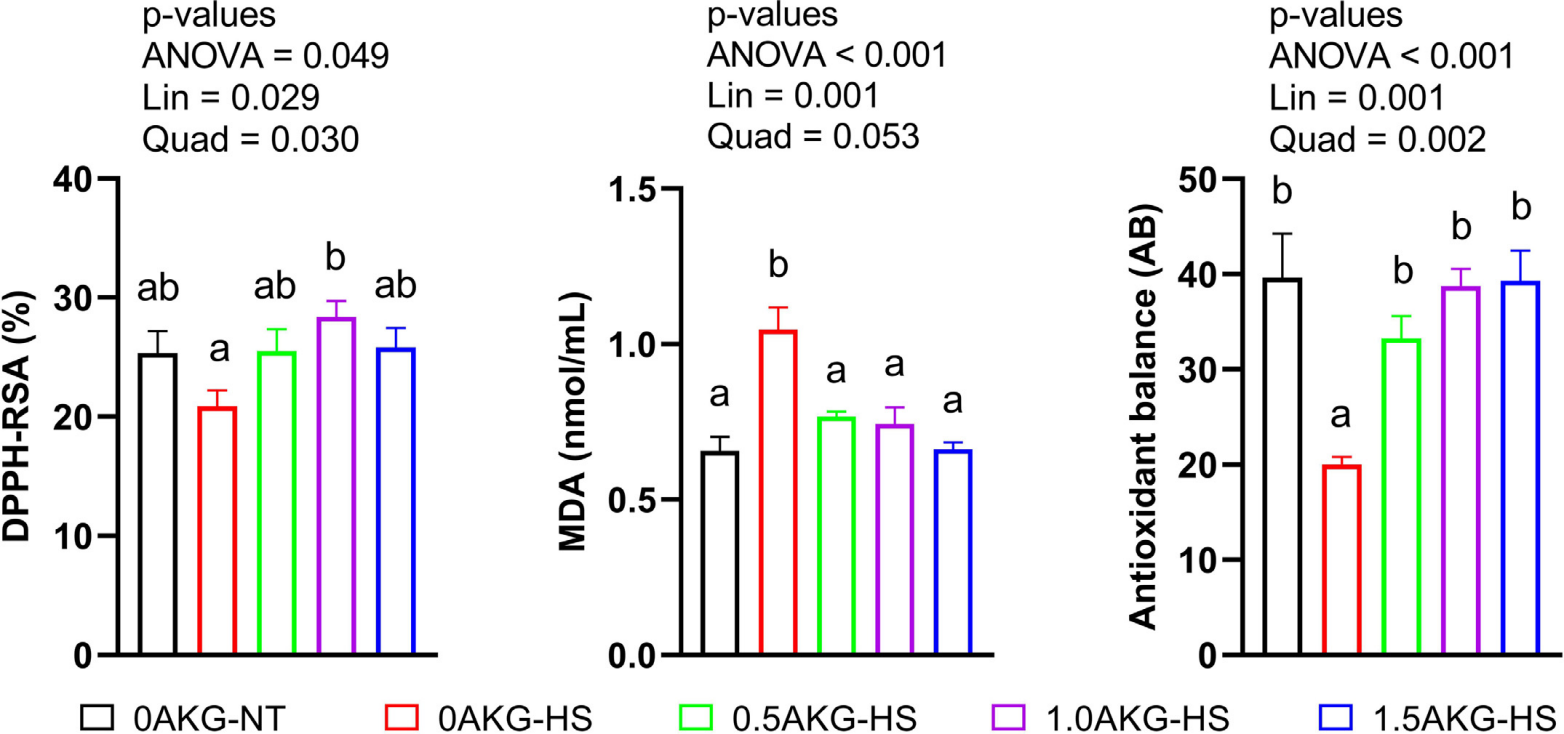
Effect of in ovo administration of a graded dosage of AKG on the plasma antioxidant markers of broilers subjected to cyclic heat stress for 6 days from days 29 to 34. At 17.5 days of incubation, eggs were injected with 0.6mL of 0% (0AKG), 0.5% (0.5AKG), 1.0% (1.0AKG), and 1.5% (1.5AKG). At the 29th day of age, birds were subjected to heat stress at 31°C ± 1°C (HS) or not (NT) and were divided into 5 groups: 0AKG-NT, 0AKG-HS, 0.5AKG-HS, 1.0AKG-HS, and 1.5AKG-HS. Data are presented as Mean ± SEM (n = 6). Means bearing different superscripts differ significantly (*P* < 0.05). Abbreviations: DPPH-RSA (%), 2,2-diphenyl-1-picrylhydrazyl free radical scavenging activity; MDA, malondialdehyde; Lin, linear effect; Quad, quadratic effect.

Total antioxidative capacity was significantly higher in 1.0AKG-HS compared to 0AKG-HS (*P* = 0.049). Moreover, significant linear (*P* = 0.029) and quadratic (*P* = 0.030) increases were seen with increasing in ovo AKG dose under HS (Figure 1). MDA concentration was significantly higher in 0AKG-HS (*P* < 0.001) compared to all the other groups. However, MDA concentration decreased linearly under HS (*P* = 0.001) with increasing amount of AKG. As a result, the decreased antioxidant balance in 0AKG-HS group was increased (*P* = 0.001) to the 0AKG-NT level in all AKG treatments.

### Hepatic mRNA Gene Regulation

**Figure 2:**
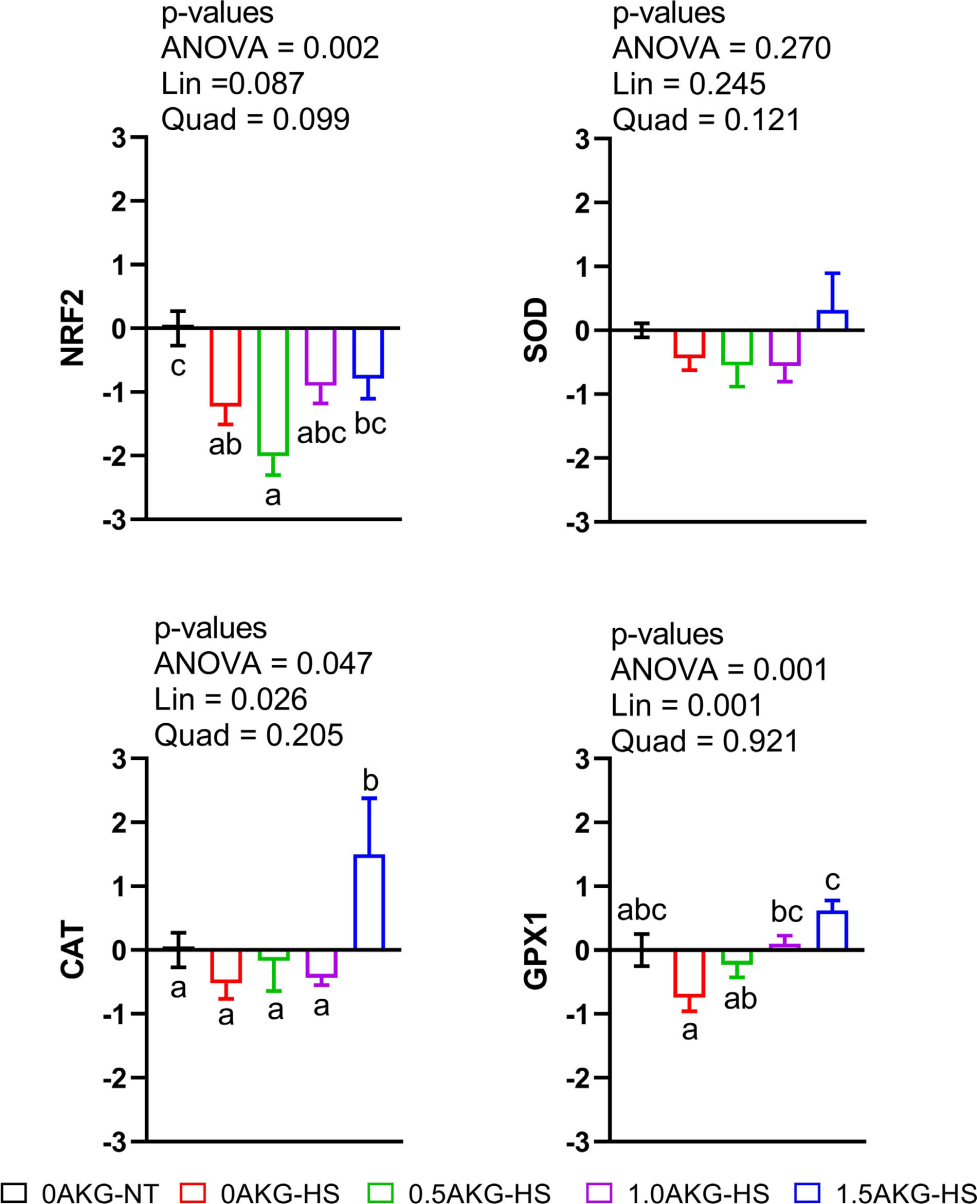
Effect of in ovo administration of a graded dosage of AKG on the hepatic mRNA gene regulation of antioxidant-related genes (A) NRF2 (B) SOD (C) CAT (D) GPX1 of broilers subjected to cyclic heat stress for 6 days from days 29 to 34. At 17.5 days of incubation, eggs were injected with 0.6 mL of 0% (0AKG), 0.5% (0.5AKG), 1% (1.0AKG), and 1.5% (1.5AKG). On the 29th day of age, birds were subjected to heat stress at 31°C ± 1°C (HS) or not (NT) and were divided into 5 groups: 0AKG-NT, 0AKG-HS, 0.5AKG-HS, 1.0AKG-HS, and 1.5AKG-HS. Data are presented as Mean ± SEM (n = 6). Means bearing different superscripts differ significantly (*P* < 0.05). Abbreviations: NRF2, nuclear factor erythroid 2–related factor; SOD, superoxide dismutase; CAT, catalase; GPX1, glutathione peroxidase 1; Lin, linear effect; Quad, quadratic effect.

Hepatic mRNA expression of NRF2 (Figure 2) was significantly downregulated (*P* = 0.002) in 0AKG-HS and 0.5AKG-HS under HS, as compared to 0AKG-NT, but was not significantly different in 1.0AKG-HS and 1.5AKG-HS. Furthermore, the relative expression of CAT was significantly higher in 1.5AKG-HS (*P* = 0.047) compared to all other treatment groups. GPX1 expression in all of AKG-HS groups did not differ from 0AKG-NT. It was linearly increased with AKG-HS compared to 0AKG-HS (*P* = 0.001), and resulted in significant upregulation in 1.5AKG-NT compared to 0AKG-HS. There were no significant changes in the expression of SOD.

**Figure 3:**
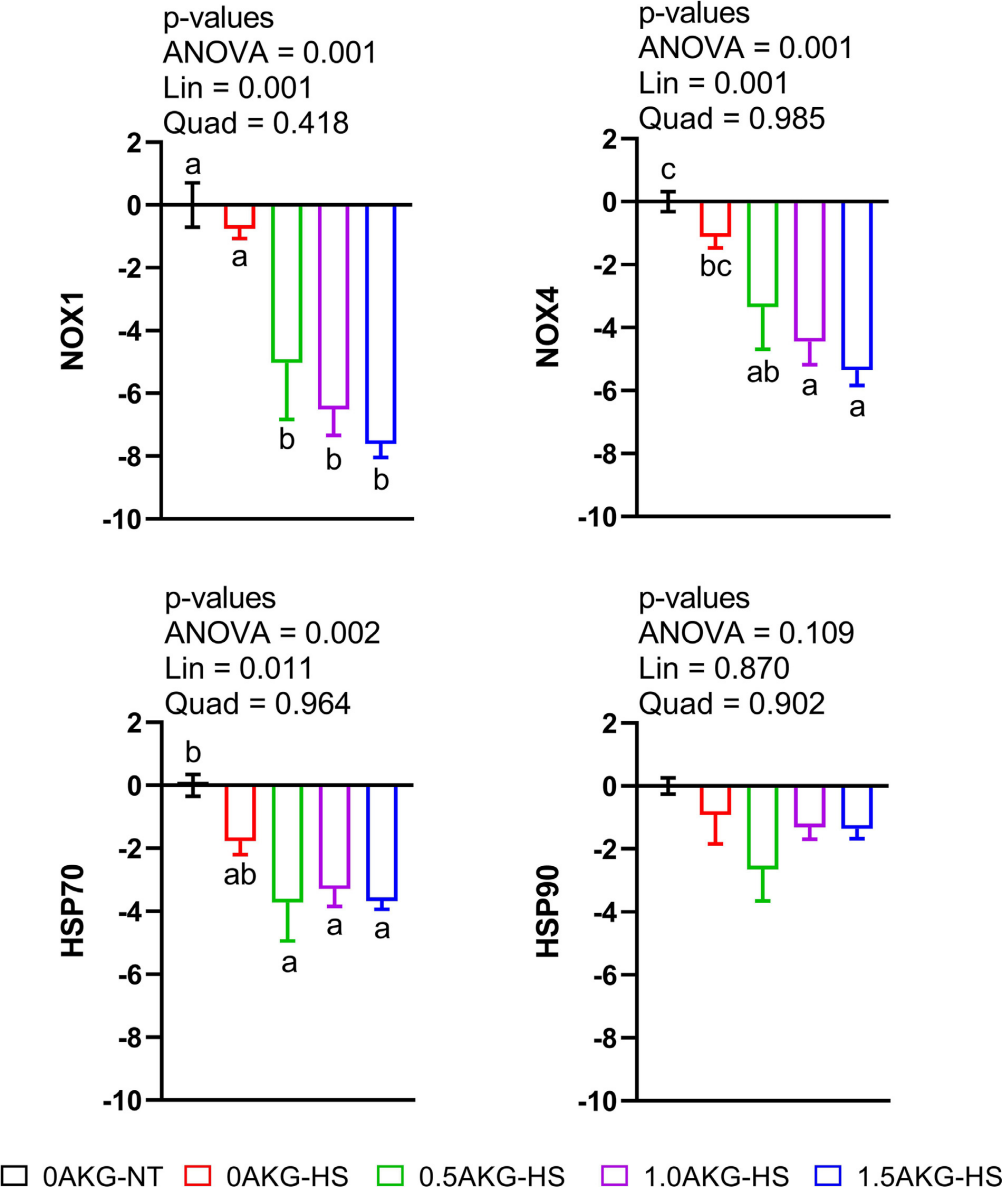
Effect of in ovo administration of a graded dosage of AKG on the hepatic mRNA gene regulation of ROS production genes (A) NOX1 (B) NOX4 and heat shock proteins (C) HSP70 (D) HSP90 of broilers subjected to cyclic heat stress for 6 days from days 29 to 34. At 17.5 days of incubation, eggs were injected with 0.6mL of 0% (0AKG), 0.5% (0.5AKG), 1% (1.0AKG), and 1.5% (1.5AKG). At 29 days of age, birds were subjected to heat stress at 31°C ± 1°C (HS) or not (NT) and were divided into 5 groups: 0AKG-NT, 0AKG-HS, 0.5AKG-HS, 1.0AKG-HS, and 1.5AKG-HS. Data are presented as Mean ± SEM (n = 6). Means bearing different superscripts differ significantly (*P* < 0.05). Abbreviations: NOX1, NADPH oxidase 1; NOX4, NADPH oxidase 4; HSP70, Heat shock protein 70; HSP90, Heat shock protein 90; Lin, linear effect; Quad, quadratic effect.

Nicotinamide adenine dinucleotide phosphate oxidase (NOX)1 and NOX4 genes were dose-dependently downregulated. Compared with 0AKG-NT and 0AKG-HS, NOX1 and NOX4 gene expression was decreased in 0.5AKG-HS, 1.0AKG-Hs, and 1.5AKG-HS (*P* = 0.001), and 1.0AKG-HS and 1.5AKG-HS (*P* = 0.001), respectively. Similarly, in ovo AKG resulted in significant downregulation in heat shock protein (HSP)70 gene (*P* = 0.011) compared to 0AKG-NT while unaffecting HSP90 gene expression (Figure 3).

**Figure 4:**
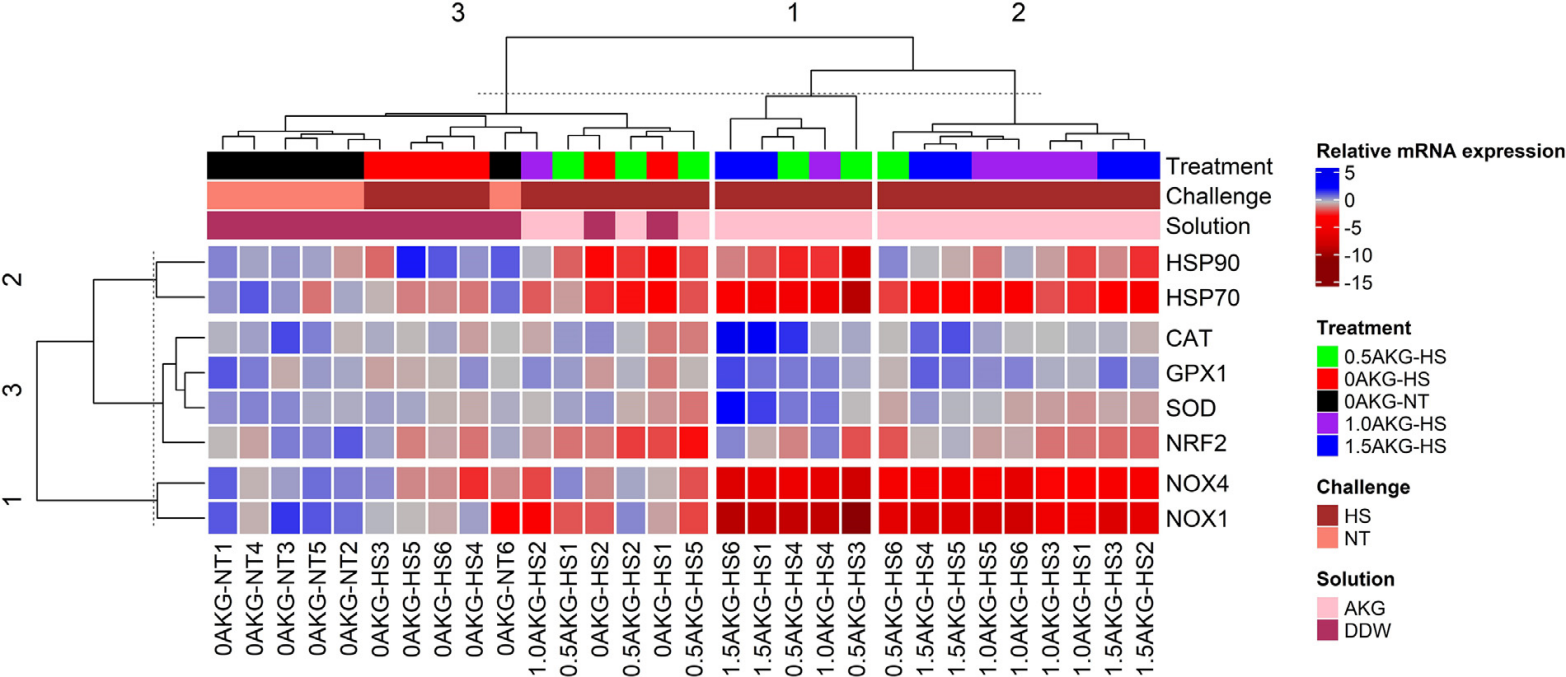
Heat map showing the hierarchical cluster of hepatic mRNA gene expression of antioxidant-related genes (HSP90, HSP70, CAT, GPX1, SOD, NRF2, NOX4, NOX1). Each row represents a gene and each column represents an experimental unit belonging to a specific treatment. At 17.5 day of incubation, eggs were given an in ovo injection of 0%, 0.5%, 1.0%, and 1.5% AKG solution prepared in DDW and subjected to heat stress (HS) during day 29 to 34 of age or not (NT) and the treatments are described as 0 AKG-NT, 0AKG-HS, 0.5 AKG-HS, 1.0 AKG-HS, and 1.5 AKG-HS, respectively. The chicks were raised in a thermoneutral environment as per standard guidelines of rearing for the entire rearing period. RT-qPCR was used for gene expression analysis. GAPDH and β-actin were used as reference genes, and the fold change (FC) of the genes was calculated as 2−ΔΔCt. The relative gene expression values were obtained as log2(FC). The tree was constructed using the package “ComplexHeatmap” of the R software version 4.0.3 (R Core Team, 2020). Abbreviations: NRF2, Nuclear factor erythroid 2-related factor; CAT, Catalase; SOD, Superoxide Dismutase; GPX1, Glutathione Peroxidase 1; HSP70, Heat Shock Protein 70; HSP90, Heat Shock Protein 90; NOX1, NADPH oxidase 1; NOX4, NADPH oxidase 4.

**Figure 5:**
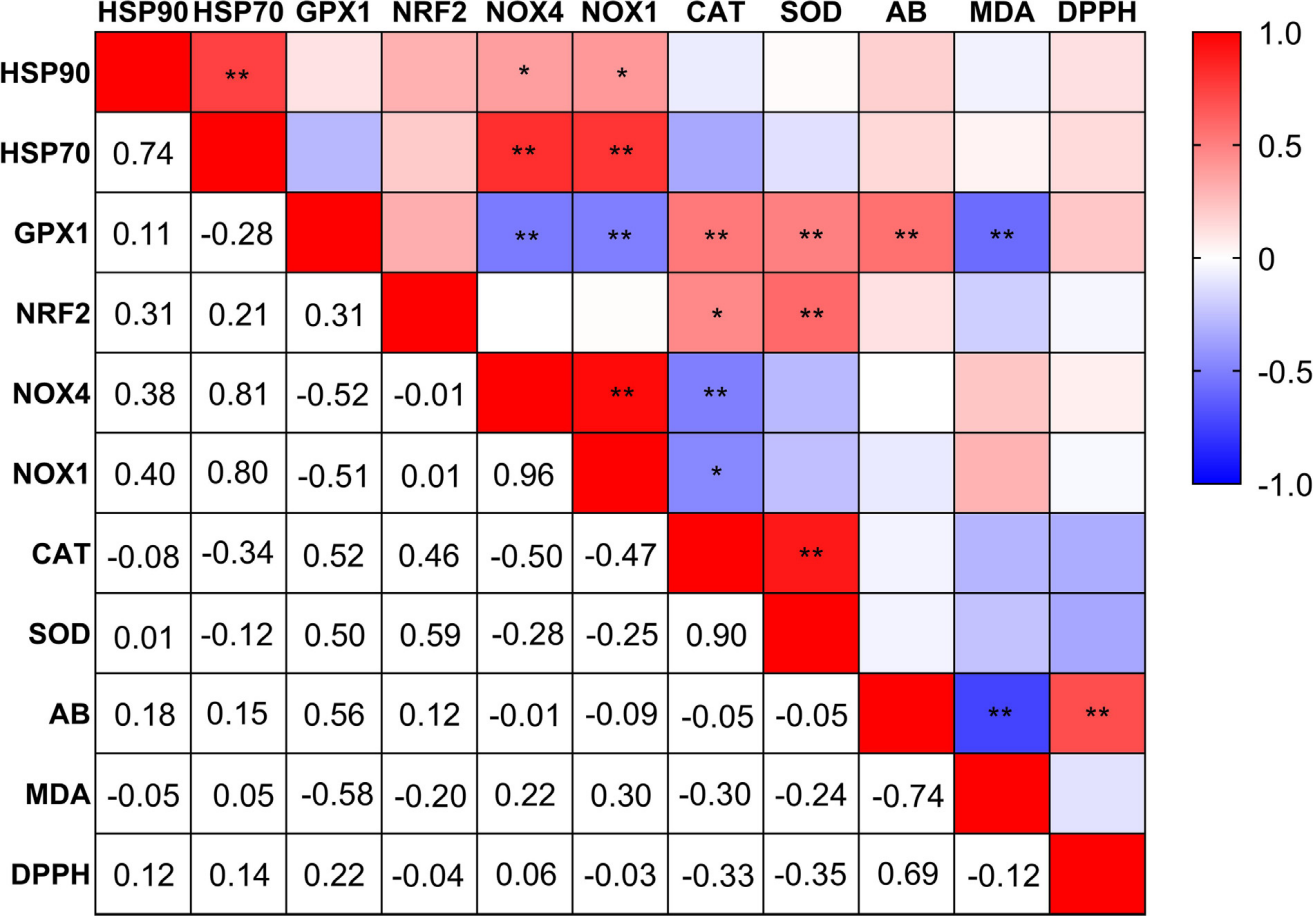
Pearson correlation heat map between hepatic gene expressions and oxidative stress markers in plasma. The red color indicates a positive correlation; the blue color indicates a negative correlation and the white color indicates no correlation. Pearson r values were calculated using the IBM SPSS Statistics for Windows software (IBM SPSS 27; IBM Corp., Armonk, NY). The heat map was realized using Graph Pad Prism 8 (GraphPad, La Jolla, CA). Abbreviations: NRF2, nuclear factor erythroid 2-related factor; SOD, superoxide dismutase; CAT, catalase; GPX1, glutathione peroxidase 1; HSP70, Heat Shock Protein 70; HSP90, Heat Shock Protein 90; NOX1, NADPH oxidase 1; NOX4, NADPH oxidase 4; AB, antioxidant balance; MDA, malondialdehyde; DPPH, 2,2-diphenyl-1-picrylhydrazyl free radical scavenging activity. ** Correlation is statistically significant at the 0.01 level. * Correlation is statistically significant at the 0.05 level.

As per the Euclidian distances, the hepatic gene expression data were vertically clustered into 3 groups (Figure 4). The first and second clusters, located at the center and right of the map, respectively, were associated with in ovo AKG injection and were subjected to cyclic HS for 6 d. These individuals showed strong downregulation (red) of NOX1 and NOX4 genes. HSP70 and HSP90 genes were also strongly downregulated in the first cluster but moderately downregulated in the second cluster. Additionally, individuals in the first cluster also exhibited strong upregulation (dark blue) of 4 antioxidant-related genes: CAT, GPX1, SOD and NRF2. The third cluster, shown on the far left of the graph, was dominated by individuals injected with DDW during incubation and had individuals with intermediate regulation of genes. Furthermore, 3 horizontal clusters are seen. The first cluster being the bottommost is comprised of NOX-genes (NOX1 and NOX4) denoting their correlation. Cluster two (topmost tier) consists of HSPs showing that their expression is correlated. The middle tier, or cluster three, consists of the maximum number of genes studied and encompasses the antioxidant-related genes (CAT, GPX1, SOD, and NRF2). The genes clustered together could indicate a pattern in expression. This is further studied as Pearson's correlation (Figure 5).

Figure 5 shows Pearson's correlation between the relative regulation of different hepatic genes, MDA concentration, DPPH-RSA (%), and AB in plasma. HSP70 gene expression was highly correlated (red) with HSP90 (r = 0.74, *P* < 0.01), NOX1 (r = 0.80, *P* < 0.01), and NOX4 (r = 0.81, *P* < 0.01). Furthermore, NOX1 (r = 0.40, *P* < 0.05) and NOX4 (r = 0.38, *P* < 0.05) were positively correlated (pale red) with the expression of HSP90. The overall expression of GPX1 showed a high positive correlation with the expression of CAT (r = 0.52, *P* < 0.01), SOD (r = 0.50, *P* < 0.01), and AB in plasma (r = 0.56, *P* < 0.01). On the other hand, a high negative correlation (blue) of GPX1 expression was noticed with NOX1 (r = −0.51, *P* < 0.05), NOX4 (r = −0.52, *P* < 0.05) and MDA concentration of plasma (r = −0.58, *P* < 0.05). The relative gene expression of NRF2 was significantly correlated with CAT (r = 0.46, *P* < 0.05) and SOD (r = 0.59, *P* < 0.01). There was a high positive correlation between NOX1 and NOX4 (r = 0.96, *P* < 0.01), whereas a negative correlation was detected between CAT and NOX4 (r = −0.50, *P* < 0.01) and between CAT and NOX1 (r = −0.47, *P* < 0.05). SOD and CAT gene expression also showed a high correlation (r = 0.90, *P* < 0.01). Finally, in plasma, AB showed a strong positive correlation with DPPH (r = 0.69, *P* < 0.01), but a strong negative correlation with MDA concentration (r = −0.74, *P* < 0.01).

**Figure 6:**
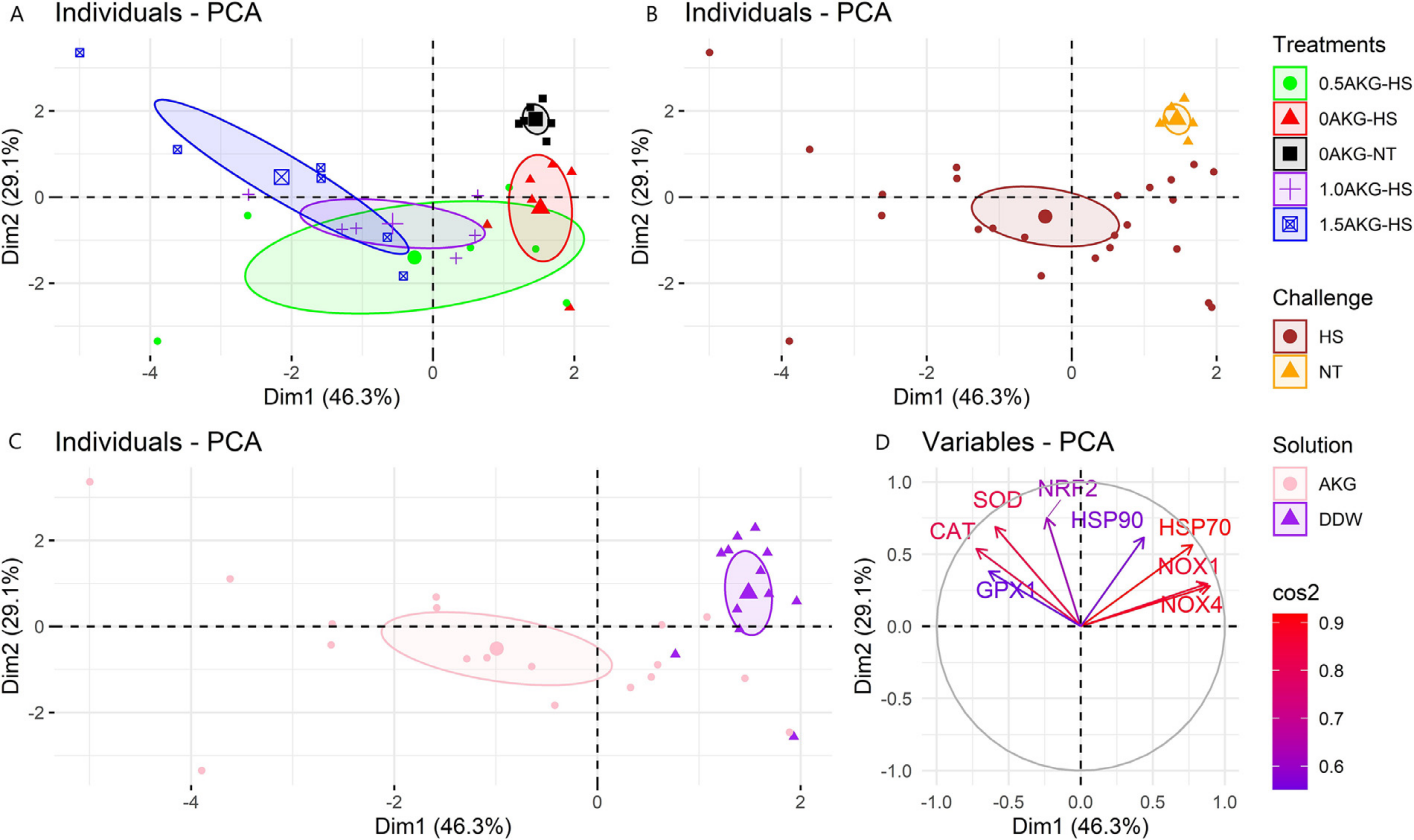
Principal component analysis (PCA) plot of individuals and variables. An individual refers to a sampled bird per treatment while a variable is a biological parameter analyzed. The individuals have been colored according to the treatments (A), challenge (B), and solution injected (C). The variables have been colored based on their squared cosine (D). The PCA was executed in R using the package “FactoMineR.” Abbreviations: NRF2, nuclear factor erythroid 2-related factor; SOD, superoxide dismutase; CAT, catalase; GPX1, glutathione peroxidase 1; HSP 70, heat shock protein 70; HSP90, heat shock protein 90; NOX1, NADPH oxidase 1; NOX4, NADPH oxidase 4; AB, antioxidant balance; MDA, malondialdehyde; DPPH, 2,2-diphenyl-1-picrylhydrazyl free radical scavenging activity; Dim1, dimension 1; Dim2, dimension 2.

Hepatic mRNA gene expression of various enzymes was used to construct PCA plots, where the 2 dimensions represent about 75% of the dataset variability. Furthermore, better contrast in the results was obtained by assigning different colors for each treatment (Figure 6A), HS challenge (Figure 6B), and in ovo feeding (Figure 6C). Figure 6D shows the different variables colored based on their squared cosine. Figure 6D shows that CAT, GPX1, HSP70, NOX1, and NOX4 were correlated with the first dimension, whereas NRF2, HSP90, and SOD were correlated with the second dimension. Figure 6C demonstrates a clear separation in the first dimension between in ovo AKG (pink) and DDW (purple). AKG injection resulted in high expression of GPX1 and CAT whereas DDW resulted in high expression of HSP70, NOX1, and NOX4 (see also Figure 2, Figure 3). Figure 6B also shows a discernible contrast between birds reared under HS (brown) and NT (orange), indicating that antioxidant enzymes are much more likely to be activated to combat adverse effects under stress conditions. 1.5AKG-HS represented the most strongly upregulated antioxidative enzymes (mostly situated leftmost in Figure 6A).

## DISCUSSION

In the present study, in ovo feeding of AKG significantly downregulated NOX and HSP70 gene expression linearly while significantly improving CAT and GPX1 gene expression in the liver of broilers under HS. This suggests a protective role of AKG as an antioxidant against oxidative damage in the liver. HS affects oxidative stress in organisms by perturbing the equipollence of reactive oxygen species (ROS) generated and obliterated from the body (Nawab et al., 2018). As such, elevated ambient temperatures cause malefic oxidative status in broilers (Lin et al., 2000; Altan et al., 2003; Mahmoud and Edens, 2003; Mujahid et al., 2007; Ncho et al., 2021a; Ncho et al., 2021c). During oxidative stress, NOXs were usually highly expressed to react with oxygen molecules and convert them to O_2_^−^ (Surai et al., 2019). Hence, we evaluated the effect of cyclic HS on these oxidative stress-related enzymes in the liver. In the current study, NOX1 and NOX4 were linearly downregulated in the heat-stressed birds with increasing amounts of in ovo AKG (Figure 3). This downregulation was further accompanied by linear downregulation of HSP70 and upregulation of GPX1 and CAT gene expression (Figure 2). A strong correlation was found between NOX enzymes and HSP genes in the current study (Figure 5). Further, the hierarchically clustered heat map (Figure 4) for various gene expressions showed that in ovo injection of AKG downregulated the NOXs and HSPs while simultaneously upregulating the antioxidant-related genes (see Figure 4 clusters 1 and 2). A previous study on hybrid sturgeon fishes demonstrated a protective role of dietary 1% AKG during ammonia-N stress by improving the activity of antioxidant enzymes and altering the hepatic gene expression of HSP70 and HSP90 (Wang et al., 2017). Moreover, AKG also prevented the lipopolysaccharide-induced increase in HSP70 in piglet enterocytes (Hou et al., 2010).

Dietary AKG significantly increased GPX, CAT, and SOD activities and glutathione concentrations in the blood of grass carps (Lin et al., 2020), indicating an improved antioxidant capacity. These enzymatic functions are important to protect the lipid peroxidation of cell membranes damage caused due ROS (Anthonymuthu et al., 2016). The property of AKG was also highlighted in rats suffering from hepatotoxicity and hyper-ammonia, induced by alcohol and ammonium acetate respectively (Velvizhi et al., 2002a; Velvizhi et al., 2002b). AKG reacts with ammonia to form glutamine (Liu et al., 2018). Glutamine is well known to mitigate HS in poultry via several mechanism including NRF2 activation (Hu et al., 2020b) and altering antioxidant related gene expression (Hu et al., 2020a). Moreover, glutamine has been extensively reviewed as an oxidative stress alleviating compound in poultry (Ncho et al., 2023b). Several mice models depict that knocking out of NRF2 gene increased the predisposition of animals towards oxidative injury (Kensler et al., 2007; Walters et al., 2008; Klaassen and Reisman, 2010; Ma and He, 2012). Hence, in the body system, NRF2 incepts the actuation of the key enzymes responsible for ROS scavenging (Ma, 2013). In our previous study (Gupta et al., 2022), we demonstrated that in ovo feeding of AKG improved the hepatic expression of NRF2 and antioxidant status of day-old chicks. The possible mechanism could be explained via glutamine synthesis which is known to activate the NRF2 pathway (Wang et al., 2015). Further, effect of AKG on the activity of primary antioxidative enzymes in various animal models have been well-reviewed (Liu et al., 2018).

To further strengthen our hypothesis, we explored the plasma antioxidative status of broilers. Two common oxidative stress markers in cells are (a) DPPH-RSA, a marker of TAC, and (b) MDA concentration, a marker of lipid peroxidation (Janaszewska and Bartosz, 2002). In the current study, in ovo AKG significantly improved TAC while decreasing MDA concentrations, thus concomitantly increasing antioxidant balance in plasma. AKG, as a key compound in Kreb's cycle is converted to succinate while simultaneously decomposing H_2_O_2_ (Kurhaluk, 2024). AKG acted as an H_2_O_2_ scavenger, in vitro by converting it into water and CO_2_ and simultaneously getting oxidized to succinate (Long and Halliwell, 2011). AKG played a role in the non-enzymatic oxidative decarboxylation by producing succinate during the decomposition of hydrogen peroxide in rats subjected to oxidative stress induced by ammonium acetate (Velvizhi et al., 2002a). This property was further illustrated in cherry Valley ducks where a dietary supplementation of 0.5% AKG significantly reduced the hepatic H_2_O_2_ concentration (Guo et al., 2017). Further, glutathione is also a known ROS scavenger and can be formed via glutamate metabolism (Zdzisińska et al., 2017). As AKG serves as a precursor to glutamine, it ultimately has a role in the biosynthesis of glutathione. Moreover, AKG helped in improving the total antioxidant capacity of aged mice (Niemiec et al., 2011). Hence, AKG ultimately acts as an antioxidative agent helping improve the antioxidant status along with decreasing the oxidative damage in birds via enzymatic and non-enzymatic pathways.

Birds can maintain their core body temperature even as ambient temperature changes (Kadono and Besch, 1978). However, exposure to higher temperatures for longer duration can lead to elevating body temperature in birds (Ncho et al., 2022b). Feed consumption, FCR and body weight gain in broilers were negatively correlated to body temperature (Cooper and Washburn, 1998). As HS leads to an increase in RT (He et al., 2019; Ncho et al., 2022b), it can be assessed as a thermotolerance marker (Chen et al., 2013). In our study, all the birds subjected to HS showed an elevated RT (Table 4). This is in accordance with the previous studies where acute (Sandercock et al., 2001) and chronic (Cooper and Washburn, 1998) heat stress led to an increase in the core body temperature of the birds. However, the current study shows that in ovo AKG resulted in an enfeebled rise in the RT compared to DDW injected group. Similarly, an attenuated rise in the core body temperature of mice was reported under acute HS conditions when supplemented with dietary glutamine (Soares et al., 2014). It is rudimentary to mention that AKG is a precursor of glutamine. We speculate that AKG might affect the RT of broilers via glutamine metabolism. It is well known that prostaglandins and interleukins are capable of inducing hyperthermia in broilers (Macari et al., 1993). In rats, glutamine influences the leukotriene and prostaglandin metabolism and downturns the cytokines circulation in the blood (Cruzat et al., 2010; Singleton and Wischmeyer, 2007). Further, it was evidenced that when monocytes are exposed to hyperthermia at 41°C, the cells deprived of glutamine displayed reduced thermoresistance (Pollheimer et al., 2005). Hence, AKG might participate in protective hypothermic mechanisms via glutamine under HS. Further studies relating to the blood eicosanoid lipid mediators in broilers could lead us to interesting results. Although the exact pathway of thermoregulation via AKG is still unclear, the present results indicate that in ovo AKG imparts superior thermotolerance in birds in regards to core body temperature compared to the DDW injected group.

Under HS, plasma metabolites could be indicators of various metabolic processes in the body (Tomonaga et al., 2018). Birds lack sweat glands leading low capacity to tolerate heat. Hence, amidst other behavioral adaptations, they rely on panting for heat loss (Ensminger et al., 1990). This leads to an excessive loss of carbon dioxide (CO_2_) from the body (Dawson, 1982; Kassim and Sykes, 1982), increasing blood alkalinity. As a result, it reduces the ionization of calcium in the blood (Allahverdi et al., 2013) leading to respiratory alkalosis in birds (Calder Jr and Schmidt-Nielsen, 1966). Moreover, at high temperatures, calcium uptake via duodenal epithelial cells is reduced (Mahmoud et al., 1996). The plasma calcium level is of greater importance in the layer birds, as this can significantly determine the eggshell quality (Marder and Arad, 1989). Plasma calcium concentration was quadratically increased in the current study, with the highest being in 1% AKG-HS group. Dietary supplementation of AKG improved collagen synthesis and immature collagen content in the bones of 30-wk layer hens (Tomaszewska et al., 2020). As laying hens use calcium from the medullary bone during the laying period, resulting in weakened medullary bone, dietary AKG can help improve bone mass and thickness. In other poultry species such as turkeys, AKG, gavage-fed directly into the crop, improved weight, mean relative wall thickness, strength, and volumetric density of the bones (Tatara et al., 2005). Hence, the results of the current study fall in line with the previous studies. Thus, in ovo feeding of AKG might also help improve calcium metabolism for bone health in broilers.

Increased panting resulted in reduced feed intake in birds (Mack et al., 2013). In the current study, although ΔBW (%) and ADG of all treatments with HS were lower than those with NT, ADFI did not differ significantly between treatments irrespective of whether cyclic HS was applied. One possible explanation for this could be that birds subjected to cyclic HS consumed feed during the recovery period (the time of day when they were not under cyclic HS) and replenished their energy. However, a significantly reduced ADG in all the groups under cyclic HS indicates that birds utilized energy to lose heat to the surroundings. Metabolic heat production in birds is highly dependent on ambient temperature (Nascimento et al., 2017). This was reflected as poor FCR across the treatments under cyclic HS as the energy consumed via feed was mostly utilized for heat loss by the broilers. Additionally, dietary supplementation of 1% AKG in layers reared under thermoneutral temperature had no differential effect on BW and FI (Tomaszewska et al., 2020). On the other hand, in other animal species, such as hybrid sturgeons, fed on high protein concentrate diet, dietary AKG improved weight gain ratios (Wang et al., 2016). Supplementation of a 7.5 g/kg diet in grass carps led to significantly improved growth performance as compared to control group (Lin et al., 2020). Glutamine, which can be interconverted to AKG and vice versa showed variable results in studies conducted on poultry over time. Dietary supplementation of 1% glutamine improved broilers weight at 6 wk of age (Soltan, 2009). However, other studies did not report differential effects on growth performances of broilers receiving glutamine supplementation (Maiorka et al., 2000; Sakamoto et al., 2006). Together, it can be ascertained that AKG does not have an adverse effect on growth in broilers. In ovo feeding of AKG did not improve or deter growth performance in broilers, suggesting that cyclic HS appears to be the driving cause for poor growth in this study.

Summarizing, in ovo feeding of AKG did not improve or deter the growth performances of broilers during HS. However, the cellular oxidative stress induced by HS can be mitigated by in ovo AKG, which resulted in a better plasma antioxidant capacity, a linear increase in DPPH-RSA (%) and AB while subsequent decrease in MDA concentration of plasma, as compared to 0AKG group subjected to HS. The expression of liver antioxidant-related genes CAT and GPX1 linearly increased in the AKG group compared to the 0AKG group under HS. Concomitantly, NOX1 and NOX4 showed significant downregulation linearly with increasing AKG dosage as compared to 0AKG-HS. Moreover, groups fed in ovo AKG showed better hepatic antioxidant status in contrast to DDW fed group. The change in RT during HS was feeble compared to DDW injected group subjected to HS. Hence, the overall findings suggest that in ovo feeding of AKG improves hepatic and plasma antioxidant status in broilers and staggers the hike in RT during HS without affecting growth parameters.

## Appendix Supplementary materials

Image, application 1

## Footnotes

Supplementary material associated with this article can be found in the online version at doi:10.1016/j.psj.2024.103749.
