# Supplementary material for: In ovo feeding of α-ketoglutaric acid improves hepatic antioxidant-gene expression, plasma antioxidant activities and decreases body temperature without affecting broiler body weight under cyclic heat stress
Source: Poult Sci. 2024 Apr 9;103(6):103749. doi: 10.1016/j.psj.2024.103749 (PMC11066556; doi:10.1016/j.psj.2024.103749)
Supplement: Supplementary file 1 [file mmc1.docx]

Supplementary table 1. Feed composition and nutrient levels of the diet^*^

| Ingredients (%) | Starter | Grower | Finisher |
| --- | --- | --- | --- |
|  | 0-7 days | 8-21 days | 22-34 days |
| Corn | 38.97 | 45.87 | 39.68 |
| Wheat | 15 | 15 | 25 |
| Soybean meal (42.6% CP) | 32 | 25.6 | 20.6 |
| Corn gluten | 3 | 2.64 | 3 |
| Meat and bone meal | 2 | 2 | 2.5 |
| Animal fat | 4 | 3.88 | 4.54 |
| Salt | 0.25 | 0.25 | 0.25 |
| Tricalcium phosphate | 1.3 | 1.04 | 0.86 |
| Limestone | 1.26 | 1.22 | 1.26 |
| Sodium bicarbonate | 0 | 0.02 | 0 |
| L-Threonine | 0.12 | 0.16 | 0.16 |
| Lysine | 1.23 | 1.44 | 1.32 |
| D-L-Methionine | 0.33 | 0.03 | 0.03 |
| Choline chloride (50%) | 0.03 | 0.03 | 0.03 |
| Premix^#^ | 0.2 | 0.2 | 0.2 |
| Phytase | 0.05 | 0.05 | 0.05 |
| Feed additive | 0.25 | 0.25 | 0.25 |
| Anti-coccidia | 0.01 | 0.01 | 0.01 |
| Calculated nutrients |  |  |  |
| Crude protein (%) | 23 | 20.5 | 19.5 |
| Crude fat (%) | 6.31 | 6.36 | 6.9 |
| Crude fibre (%) | 3.01 | 2.8 | 2.68 |
| Crude ash (%) | 5.99 | 5.34 | 5.02 |
| Calcium (%) | 1.01 | 0.9 | 0.86 |
| Available phosphorous (%) | 0.6 | 0.53 | 0.49 |
| Digestible lysine (%) | 1.43 | 1.24 | 1.09 |
| Digestible methionine + cystine (%) | 1.07 | 0.95 | 0.86 |
| Copper (ppm) | 82.21 | 81.04 | 80.78 |
| Zinc (ppm) | 100.27 | 96.63 | 97.33 |
| Metabolizable energy (kcal/kg) | 3050 | 3150 | 3200 |

* Feed was procured from Nonghyup Feed (Seoul, Korea).

^#^ Trace minerals and vitamins provided in premix: Vitamin A, 12,000,000 IU; Vitamin D_3,_ 3,000,000 IU; Vitamin E, 40,000 IU; Vitamin K_3,_ 2,000 IU; Vitamin B_1,_ 2,000 mg; Vitamin B_2,_ 5,000 mg; Vitamin B_6,_ 3,000 mg; Vitamin B_12,_ 20 mg; Niacin, 40,000 mg; Pantothenic acid, 10,000 mg; Folic acid, 1,000 mg; Iron, 88,000 mg; Copper, 72,600 mg; Zinc, 60,000 mg; Manganese, 66,000 mg; Iodine, 990 mg; Selenium, 220 mg; Cobalt, 330 mg
